# The Effects of Temperature on the Kinematics of Rattlesnake Predatory Strikes in Both Captive and Field Environments

**DOI:** 10.1093/iob/obaa025

**Published:** 2020-10-04

**Authors:** M D Whitford, G A Freymiller, T E Higham, R W Clark

**Affiliations:** 1 Department of Biology, San Diego State University, San Diego, CA, USA; 2 Ecology Graduate Group, University of California, Davis, CA, USA; 3 Department of Evolution, Ecology, and Organismal Biology, University of California, Riverside, CA 92521, USA; 4 Chiricahua Desert Museum, Rodeo, NM, USA

## Abstract

The outcomes of predator–prey interactions between endotherms and ectotherms can be heavily influenced by environmental temperature, owing to the difference in how body temperature affects locomotor performance. However, as elastic energy storage mechanisms can allow ectotherms to maintain high levels of performance at cooler body temperatures, detailed analyses of kinematics are necessary to fully understand how changes in temperature might alter endotherm–ectotherm predator–prey interactions. Viperid snakes are widely distributed ectothermic mesopredators that interact with endotherms both as predator and prey. Although there are numerous studies on the kinematics of viper strikes, surprisingly few have analyzed how this rapid movement is affected by temperature. Here we studied the effects of temperature on the predatory strike performance of rattlesnakes (*Crotalus* spp.), abundant new world vipers, using both field and captive experimental contexts. We found that the effects of temperature on predatory strike performance are limited, with warmer snakes achieving slightly higher maximum strike acceleration, but similar maximum velocity. Our results suggest that, unlike defensive strikes to predators, rattlesnakes may not attempt to maximize strike speed when attacking prey, and thus the outcomes of predatory strikes may not be heavily influenced by changes in temperature.

## Introduction

Predator–prey interactions occur dynamically in nature over a range of environmental conditions, and the outcome of the encounter can be strongly influenced by environmental factors. For example, sparrowhawks (*Accipiter nisus*) are less effective predators of redshanks (*Tringa totanus*) during periods of high winds, as wind has a greater influence over the ability of sparrowhawks to perform highly coordinated attacks than it does on the ability of redshanks to evade (Quinn and Cresswell 2004). For interactions that involve ectothermic species, temperature will often play a prominent role in determining the outcome due to the correlation between temperature and the performance of muscle-driven movements. Due to their temperature dependency, species interactions involving ectotherms may be impacted by anthropogenic global warming (Dell et al. 2014). Indeed, a study on coral reef fish (Allan et al. 2015), found that a 3°C increase in temperature, which is well within current climate change projections (Vose et al. 2017), resulted in marked alterations to predator–prey interactions, including increased predator attack speed and increased rate of prey capture. As temperature influences, the performance of ectotherms more than endotherms, the effects of temperature on predator–prey interactions should be most pronounced in interactions that involve both an endotherm and an ectotherm (Dell et al. 2014).

The predatory performance of viperid snakes (ectotherms) hunting nocturnal small mammals (endotherms) is a prime example of how widespread such an effect could be in different ecosystems. Crotaline viperids (rattlesnakes, copperheads, and cottonmouths) are widely distributed and abundant predators in North America, and are known to prey on a variety of small mammals (Fitch 1949; Diller and Wallace 1996; Clark 2002; Reinert et al. 2011, Dugan and Hayes 2012). As crotalines typically hunt at night when temperatures are sub-optimal for ectotherms (Huey et al. 1989; Clark et al. 2016), a positive effect of increased temperature on strike performance could result in marked alterations in rates of prey capture and the subsequent consumptive effect on prey populations. When hunting, viperid snakes typically remain motionless in a stereotyped ambush coil and wait for prey to approach within striking distance (Kardong and Bels 1998; LaDuc 2002; Herrel et al. 2011; Reinert et al. 2011; Clark et al. 2016). Once potential prey are close, vipers effect a strike by rapidly straightening their body, thereby propelling their head toward the prey, while simultaneously gaping their jaws and rotating the maxillary fangs forward to bite and inject venom (Kardong and Bels 1998). Encounters with prey are rare, and viperids can wait for several days in a single ambush coil, and weeks between strike attempts (Reinert et al. 1984; Clark et al. 2016; Putman and Clark 2017). Temperature can vary widely over these periods, leading to the potential for seasonal and diel cycles of temperature to have a strong influence on hunting success by altering strike performance. However, some rapid, single-shot ectotherm movements that are similar to the predatory strike of vipers are robust to the deleterious effects of temperature, often due to the presence of elastic recoil mechanisms commonly associated with ballistic movements (Anderson and Deban 2010; Deban and Scales 2016). Young (2010) found evidence to suggest that heavy-bodied vipers may use elastic recoil when striking, as muscle activity associated with striking occurred prior to, but not during, a strike—a pattern suggesting that snakes were actively loading elastic structures. Thus, there is a possibility that large-bodied vipers can partially circumvent the deleterious effects of low body temperature on strike performance. However, several studies on the scaling relationships between strike performance and snake size indicate that larger snakes can accelerate more rapidly, and, in some species, can also attain higher maximum velocities (Herrel et al. 2011; Penning et al. 2019). The increased strike performance at larger body sizes is the result of a negative allometric relationship between head size and body size, while the dominant epaxial muscles used during a strike scale either isometrically or positively with body size.

The effect of temperature could also be minimal if snakes are not attempting to maximize velocity (i.e., performing below their physiological limits; Astley et al. 2013). The predatory strike of viperids involves a coordinated sequence of movements directed toward a relatively small target, usually in low-light environments. If free-ranging snakes prioritize accuracy over speed, for example, the link between temperature and strike velocity may be weak under natural conditions. Compared to defensive strikes (strikes toward a threatening stimulus), rattlesnakes striking prey in captivity often strike more slowly (LaDuc 2002), suggesting that they are not maximizing speed during an offensive strike.

Although surprisingly few studies have directly examined the role of temperature on snake strike behavior and kinematics, the existing research indicates that defensive strike velocity and acceleration does increase with temperature, but not to the extent that would be expected if the movement was driven purely by muscle contraction (Whitford et al. 2020). However, the kinematics of defensive strikes and predatory strikes is different (LaDuc 2002), with defensive strikes reaching higher velocities, farther distances, and having a greater percentage of the body kinematically active. Furthermore, almost all previous analyses of strike performance have been done in captive environments (but see Whitford et al. 2019b), a context in which motivational state, time in captivity, stress from being in an enclosed space, and various additional factors could have a strong effect on performance.

Here, we used high speed videography to study the effects of temperature on the predatory strikes of rattlesnakes using a combination of field and lab experiments. We also compared the performance and influence of temperature between predatory strikes and the defensive strikes filmed using a similar experimental setup in a previous study (Whitford et al. 2020). Our goals were to (1) understand the effects of temperature on predatory strike performance across different experimental contexts and (2) determine if temperature differentially influences predatory and defensive strikes. Additionally, as we noticed a pre-strike movement toward prey in our experimental trials that we had not seen in recordings of free-ranging snakes, we examine the prevalence of this movement between different experimental contexts and discuss its significance. Given that our previous study on defensives strikes found that most strike kinematics were associated with relatively low Q_10_ values (<1.6) and that previous studies (LaDuc 2002) found that predatory strikes are slower than defensive strikes, we predicted that the influence of temperature on predatory strikes would be limited in both lab and field contexts.

## Methods

In order to incorporate both ecological realism and the ability to more carefully standardize environmental factors, we recorded predatory strikes both in the field, from free-ranging individuals, and from captive individuals in the lab (see Table 1 for video examples). First, using a previously published dataset (Whitford et al. 2019b), we examined the effects of temperature on predatory strikes from free-ranging sidewinder rattlesnakes (*Crotalus cerastes*) directed at free-ranging desert kangaroo rats (*Dipodomys deserti*). As the body temperatures of snakes in this dataset typically fell between 25°C and 35°C, which is likely near the peak of their thermal performance curve (Rowe and Owings 1990; Stepp-Bolling 2012; Whitford et al. 2020), we then recorded strikes from free-ranging Mohave rattlesnakes (*Crotalus scutulatus*) toward warmed, dead lab mice, which allowed to us quantify strike velocity across a broader range of body temperatures. To examine the effects of temperature under standardized conditions, we then recorded predatory strikes from captive snakes, which allowed us to better control temperature and mitigate outside influences on strike performance. We initially attempted to use Mohave rattlesnakes in the captive experiments, but found our captive Mohave rattlesnake individuals to be uncooperative feeders in the laboratory environment. Our previous research compared the defensive strike behavior and kinematics of Mohave rattlesnakes to that of their sister species, western rattlesnakes (*Crotalus oreganus*), and found much more variation to be present between individuals within species than between the two species themselves (Whitford et al. 2020). Thus, we used our captive colony of western rattlesnakes (*C. oreganus*), which had some individuals that feed readily under experimental conditions, for the laboratory predatory experiment. All rattlesnakes used in the laboratory experiments were housed at San Diego State University and are part of a permanent collection. Captive snakes were given ad libitum water, fed a mouse (*Mus musculus*) every other week, maintained at a constant temperature of 28°C–30°C, and kept on 12 L:12 D light schedule.

**Table 1:** Video examples for the lab experiment, field experiment, and a typical strike directed at free-ranging prey

URL	Information
Video S1	A Mohave rattlesnake striking at a lab mouse in the field experiment.
Video S2	A western rattlesnake striking at a lab mouse in the lab experiment. This video also shows the snake making prestrike movements toward prey.
Video S3	A free-ranging Mohave rattlesnake striking at a pocket mouse (*Chaetodipus* sp.). The video illustrates the typical strike sequence of rattlesnakes in the wild, with no prestrike movement.

### Field strikes toward kangaroo rats

To examine the effects of temperature on predatory strikes directed at natural, free-ranging prey, we used sidewinder rattlesnake strike data from a previously published study (Whitford et al. 2019b). In our analyses, we only used strikes that accurately targeted the kangaroo rats and had reliable temperature data available. We also removed one strike as the kangaroo rat was ∼3 cm from the snake and moving toward the snake. The methods were identical to those we used in our field experiment (described below) with a few exceptions. Rather than eliciting strikes using a lab mouse, we recorded strikes toward free-ranging desert kangaroo rats. Additionally, the videos were calibrated and digitized using a 3D, rigid, metallic calibration object, Matlab, and DLTDV5 (Hedrick 2008). To extract kinematic measures, we used the *XYZ* coordinates for a point on the neck in-line with the posterior edge of the venom glands and measured strike distance as the distance between the tip of snake’s upper jaw and closest point of the prey. The neck point was digitized for all analyses, and strike distance was measured using the same methods. See Whitford et al. (2019b) for further details.

### Field strikes toward mice carcasses

To record predatory strikes from free-ranging Mohave rattlesnakes, we used radio telemetry to monitor a population of snakes in Rodeo, NM (31.889092 N, −109.030752 W) from May to August 2018. We located snakes through visual encounter surveys and implanted adults of both sexes with temperature-sensitive radio-transmitters (Reinert and Cundall 1982). After they recovered from surgery (typically within 12 h), snakes were released at the site of capture and subsequently monitored nightly. Upon relocating snakes, we recorded their body position, behavior, and body temperature. When a snake was found to be hunting in a stereotyped ambush coil (Reinert et al. 1984, Reinert et al. 2011), we positioned two synchronized Edgertronic cameras (Model SC1) and 4–6 infrared lights ∼1–2 m from the snake. The cameras were connected via an Ethernet cable to a laptop computer located ∼15 m from the snake. Once the camera setup was positioned, we waited 30 min before attempting to elicit a strike in order to account for any disturbance to the snake that might have been caused by equipment placement. During previous studies, we found that rattlesnakes will readily attempt to capture prey following the positioning of the cameras (Higham et al. 2017; Whitford et al. 2017; Freymiller et al. 2019; Whitford et al. 2019b). After the 30 min waiting period, we attempted to elicit a predatory strike by moving a dead, warmed (∼38°C) mouse carcass (*M. musculus)* in front of the snake in a manner akin to small mammal foraging movement. We used a carcass to elicit strikes because we knew from previous experiments that the rate of natural prey encounters would be prohibitively low, and our experiment required a large sample across a broad temperature gradient. The mouse was attached to a thin metal rod that extended downward, ∼1 m from the end of a pole held by the experimenter. To standardize the mouse movement, the same individual always manipulated the carcass. We moved the mouse across the snake’s field of view (perpendicular to the orientation of the head) approximately 3 times beginning at ∼15 cm, ∼10 cm, and ∼5cm. If a strike occurred, the temperature of the snake was recorded from the pulse rate of the temperature-sensitive radio transmitter. Following each strike, a rigid, metallic ruler was waved through the space occupied by the snake to allow for video calibration using Matlab and easyWand (Hedrick 2008; Theriault et al. 2014; Jackson et al. 2016). The *XYZ* coordinates for a point on the neck of the snake, in-line with the posterior edge of the venom glands, were then extracted; this point was chosen to remove any potential effects that opening the mouth would have on the kinematics of the strike.

### Captive strikes toward live mice

To record predatory strikes in the lab from western rattlesnakes, individual snakes were placed in a 50 cm (w) × 50 cm (l) × 30 cm (h) cm plywood enclosure with a transparent acrylic front wall. The enclosure was housed in a temperature regulated room. Snakes were left in the enclosure overnight and given at least 12 h to acclimate to the enclosure and the room temperature. Once acclimated, a mirror was placed at a 45° angle on top of the enclosure, such that the camera could record a top-down view of the snake, providing a second viewing angle of the strike. A lab mouse of known weight (the typical food provided for these long-term captive snakes) was then placed inside a custom built device to allow for remote releasing of the mouse into the enclosure. We used short wavelength infrared lighting not visible to either the mouse or the snake to illuminate the enclosure (Goris 2011), and positioned a single Edgertronic (Model SC1) high speed camera recording at 250 fps and 1/1000 shutter speed for 10 s to record the strike through the transparent side of the enclosure (head-on view) and through the mirror (top-down view), simultaneously; thus, one camera provided two different perspectives of the strike. An additional camera (Sony Handycam) recorded continuously throughout the experiment to document details outside the time frame of the high-speed recording. Following the positioning of the mirror and the mouse, the room lights were turned off (i.e., no visible light was available in the room), so that the available light was similar between strikes recorded in the lab versus in the field. We then waited 30 min before releasing the mouse. Once the mouse was released, we gave the snake ∼1 h to strike; if the snake had not stuck within the hour, it was excluded from the study. If the snake struck the mouse, it was allowed to then consume it. Following the trial, we immediately recorded the cloacal temperature of the snake, and then measured the mass and length of the snake. A three-dimensional calibration object was then placed in the enclosure to calibrate the space occupied by the snake and mouse during the strike. All videos were calibrated and digitized using Matlab and DLTDV5 (Hedrick 2008). The *XYZ* coordinates for the same neck point used in the field strikes were extracted. One strike was recorded per individual snake at both 20°C and 30°C; thus, all strikes were paired across temperature treatments. If a snake did not strike in one of the treatments, it was removed from the study. At least 2 weeks were given between treatments for each snake.

### Comparison to defensive strikes

To compare the kinematics of predatory strikes to defensive strikes, we used data for defensive strikes recorded and analyzed in Whitford et al. (2020). Briefly, using western rattlesnakes, three strikes were recorded for each snake at every 5°C increment from 15°C to 35°C. We elicited snakes to strike using a balloon and extracted 2D *XY* coordinates (for the same point on the neck) using a grid placed immediately behind the snakes and perpendicular to the camera. To create a dataset comparable to the lab predatory strikes from the current experiment, we removed all strikes from the defensive strike dataset except the first strike for each snake in the 20°C and 30° treatments; thus, this subset of the defensive strike dataset recorded in captivity mirrors that of the lab predatory strike dataset we generated here.

### Pre-strike head movements

To assess the degree to which pre-strike head movements depend on context, we reviewed video recordings of 84 strikes filmed for previous studies (Clark 2006; Barbour and Clark 2012a, 2012b; Clark et al. 2016; Higham et al. 2017; Whitford et al. 2019b) of free-ranging snakes hunting natural prey, as well as strikes recorded for both our field and lab experiment. In addition to western rattlesnakes, Mohave rattlesnakes, and sidewinder rattlesnakes, this dataset included strikes from red diamond rattlesnakes (*Crotalus ruber*), and timber rattlesnakes (*C. horridus*). For each strike, we extracted whether snakes moved their head toward prey prior to striking (binary “yes” or “no”).

### Statistical analyses

To extract the kinematic measures of interest, we used RStudio and the package “signal” to apply a 50 Hz low pass, Butterworth filter to the *XYZ* coordinates for each strike. We then extracted maximum velocity and acceleration from the filtered data. We calculated strike distance as the distance between the tip of the snake’s upper jaw and the lab mouse for the frame in which the strike was initiated. For our analyses, we log_10_-transformed the dependent variables, as temperature and biological rates often illustrate an exponential relationship.

We first analyzed field (both toward natural prey and lab mice) and lab predatory strikes separately using generalized linear mixed models and the package “lme4” (Bates et al. 2015), with either maximum velocity, maximum acceleration, or strike distance as dependent variables. We included cloacal temperature and strike distance (except in models assessing strike distance) as fixed effects, and snake ID as a random effect. For each model, we also calculated a temperature coefficient (Q_10_) by taking the antilogarithm of the partial regression coefficient for temperature multiplied by 10 (Deban and Lappin 2011; Deban and Scales 2016). To compare lab predatory and defensive strikes, we constructed two generalized linear mixed models with either strike velocity, strike acceleration, or strike distance as dependent variables. As predictor variables, we included strike distance (except in the model assessing strike distance), type of strike (binary: “predatory” or “defensive”), temperature treatment (binary: “20°C” or “30°C”), and the interaction between type of strike and temperature treatment.

To analyze the head movement data, we performed a chi-squared test that compared the proportion of strikes where snakes moved toward prey between the three experiment contexts (lab, field with lab mouse carcass, and field toward natural prey). As some snakes contributed more than one strike to the dataset, and occasionally to different experimental contexts, the strikes are not all independent individuals. To correct for nonindependence, we conducted a second chi-squared test but included only one randomly selected strike per snake.

## Results

### Field strikes toward kangaroo rats

We used 15 sidewinder rattlesnake strikes directed toward desert kangaroo rats recorded for Whitford et al. (2019b). The average body temperature was 29.6°C and ranged from 18.8°C to 36.6°C. The average maximum strike velocity and acceleration was 2.6 m s^−1^ (0.89–3.97) and 113.3 m s^−2^ (35.80–183.22), respectively. The average distance to the kangaroo rat at the initiation of the strike was 15.1 cm (5.65–24.05). We found no effect of temperature on maximum strike velocity (Est. = −0.015, SE = 0.009, *P* = 0.13; Q_10_ = 0.71), maximum strike acceleration (Est. = 0.002, SE = 0.011, *P* = 0.87; Q_10_ = 1.05), or strike distance (Est. = 0.017, SE = 0.011, *P* = 0.15; Q_10_ = 1.50). However, we did find a significant positive effect of strike distance on maximum strike velocity (Est. = 0.021, SE = 0.006, *P* = 0.006), but not maximum strike acceleration (Est. = 0.012, SE = 0.008, *P* = 0.16).

### Field strikes toward mice carcasses

**Fig. 1:**
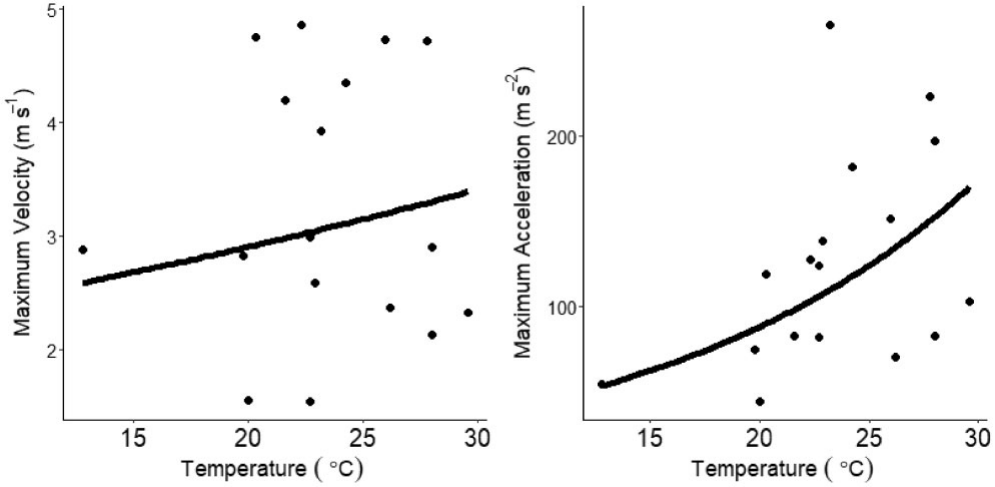
Scatterplots of maximum strike velocity and maximum strike acceleration for Mojave rattlesnakes in the field experiment. Regression lines are back–transformed predictions from the mixed models. Temperature had a statistically significant effect on maximum acceleration, but not maximum velocity.

In our field experiment using Mohave rattlesnakes, we recorded a total of 17 strikes from 10 individuals (1–3 strikes per snake). The average body temperature of snakes was 23.4°C and ranged from 12.8°C to 29.6°C. The average maximum strike velocity and acceleration was 3.27 m s^−1^ (1.54–4.86) and 125.00 m s^−2^ (44.42–265.36), respectively. On average, snakes struck at the mouse when it was 18.5 cm (4.6–43.7) away. We found no effect of temperature on strike distance or maximum strike velocity (Fig. 1; Table 2**)**. Body temperature, however, was positively correlated with maximum strike acceleration. Additionally, maximum strike velocity was positively correlated with strike distance, whereas strike acceleration was not correlated with strike distance. Similarly, the Q_10_ values for maximum strike velocity (Q_10_ = 1.17) and strike distance (Q_10_ = 0.75) indicate a minimal effect of temperature, while, maximum strike acceleration (Q_10_ = 1.99) was found to increase with temperature. However, strike distance and body temperature together only explained ∼34% (marginal *R*^2^ = 0.34, conditional *R*^2^ = 0.34) of the variation in maximum strike acceleration and body temperature alone only explained ∼23%. Thus, temperature differences could not account for most of the variation in maximum strike acceleration.

**Table 2:** Results of generalized linear mixed models assessing of the effects of strike distance and temperature on the strike performance of field predatory strikes

Variable	Est.	SE	*P*-value
Max. velocity			
Temperature	0.007	0.007	0.33
Strike distance	**0.011**	**0.002**	**0.0004**
Max. acceleration			
Temperature	**0.03**	**0.012**	**0.02**
Strike distance	0.007	0.004	0.117
Strike distance			
Temperature	−0.012	0.017	0.486

Dependent variables are left justified and the predictor variables are right justified. Bolded rows indicate statistically significant variables.

### Captive strikes toward mice

We recorded predatory strikes in captivity at 20°C and 30°C from 15 western rattlesnakes (Supplementary Fig. S1**)**. For the 20°C and 30°C treatments, average maximum strike velocity was 2.21 m s^−1^ (1.62–2.93) and 2.42 m s^−1^ (1.00–4.44), and average maximum strike acceleration was 74.45 m s^−2^ (44.66–99.50) and 93.00 m s^−2^ (41.04–132.09), respectively (Table 3). Strike distances were similar in both temperature treatments. In the 20°C treatment, average strike distance was 14.1 cm (3.5–30.3 cm), and, in the 30°C treatment, average strike distance was 13.9 cm (3.2–25.5). We found no effect of temperature on maximum strike velocity (Q_10_ = 1.09) or strike distance (Q_10_ = 0.89), but we did find a positive effect of temperature on strike acceleration (Q_10_ = 1.24; marginal *R*^2^ = 0.34; Table 4). On average, maximum strike acceleration increased by 18.55 m s^−2^ from the 20°C treatment to the 30°C treatment.

**Table 3:** Summary statistics for predatory and defensive strikes recorded in the lab

Type	Treatment	Max. velocity (m s^−1^)	Max. Acceleration (m s^−2^)	Strike Distance (cm)
Defensive	20°C	3.30 (2.31–4.41)	89.08 (59.68–134.96)	16.31 (7.60–34.10)
	30°C	3.77 (2.73–5.09)	104.56 (77.46–146.43)	16.98 (7.00–30.40)
Predatory	20°C	2.21 (1.62–2.93)	74.45 (44.66–99.50)	14.08 (3.50–30.37)
	30°C	2.42 (1.00–4.44)	93.00 (41.04–132.09)	13.90 (3.16–25.47)

Values are mean (min-max).

**Table 4:** Results of generalized linear mixed models assessing of the effects of strike distance and temperature on the strike performance of laboratory predatory strikes

Variable	Est.	SE	*P*-value
Max. Velocity			
Temperature	0.004	0.004	0.34
Strike distance	0.002	0.003	0.52
Max. acceleration			
Temperature	**0.011**	**0.002**	**0.002**
Strike distance	0.002	0.002	0.39
Strike distance			
Temperature	−0.005	0.01	0.62

Dependent variables are left justified and the predictor variables are right justified. Bolded rows indicate statistically significant variables.

### Comparison to defensive strikes

**Fig. 2:**
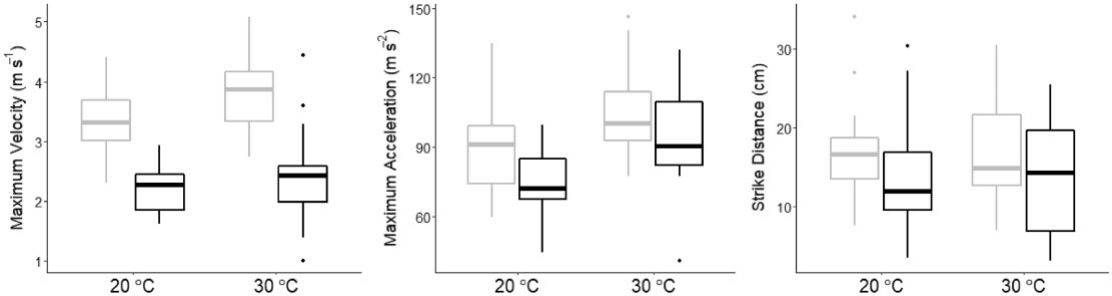
Boxplots of maximum strike velocity, maximum strike acceleration, and strike distance for both the lab defensive (gray) and lab predatory (black) strikes of western rattlesnakes in the 20°C and 30°C treatments. Defensive strikes had significantly greater maximum velocity, maximum acceleration, and distance.

When comparing predatory and defensive strikes, similar to the results of previous studies (LaDuc 2002), we found that strike distance, maximum velocity, and maximum acceleration were greater for defensive strikes than predatory strikes (Fig. 2; Table 5). In both temperature treatments, defensive strike velocity was ∼1.5 times greater than predatory strike velocity, while defensive strike acceleration was ∼1.2 times greater than predatory strike acceleration. We found no effect of the interaction between temperature and the type of strike for strike velocity, acceleration, or distance. Similar to our other analyses, we found a positive correlation between strike distance and velocity but not between distance and acceleration.

**Table 5:** ANOVA tables for the generalized linear mixed models assessing differences in strike performance between lab predatory and defensive strikes

Variable	*F*-value	*P*-value
Max. velocity		
Type	**91.35**	**<0.0001**
Temp. treatment	**9.88**	**0.002**
Strike distance	**6.1**	**0.02**
Type: temp. treatment	0.09	0.76
Max. acceleration		
Type	**8.09**	**0.006**
Temp. treatment	**26.99**	**<0.0001**
Strike distance	0.004	0.95
Type: temp. treatment	1.41	0.24
Strike distance		
Type	**5.48**	**0.022**
Temp. treatment	0.13	0.72
Type: temp. treatment	0.24	0.62

Dependent variables are left justified and the predictor variables are right justified. Bolded rows indicate statistically significant variables.

### Pre-strike head movement

**Fig. 3:**
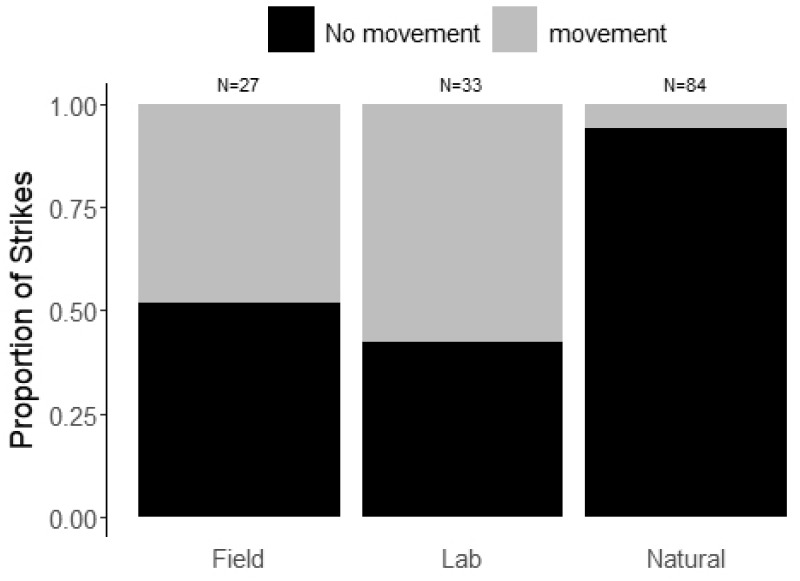
The frequency of strikes that involved prestrike movement toward the prey in our field experiment, lab experiment, and strikes directed at natural free-ranging prey. Natural strikes that included a prestrike movement toward prey included two instances from timber rattlesnakes, two from western rattlesnakes, and one from a sidewinder rattlesnake.

Our field and captive experimental recordings frequently documented snakes slowly moving their head out of their coil and toward the prey a few seconds before launching a strike (Supplementary Video S2), a behavior we had not seen in our field studies of strikes toward free ranging prey. To quantify differences in the occurrence of pre-strike head movement between different experimental contexts (natural, lab, and field), we analyzed 135 strike recordings. The dataset included 84 strikes between free-ranging snakes and natural prey, including 34 sidewinder rattlesnakes (*C. cerastes*), 21 timber rattlesnakes (*C. horridus*), 12 western rattlesnakes (*C. oreganus*), 4 red diamond rattlesnakes (*C. ruber)*, and 13 Mohave rattlesnakes (*C. scutulatus*). The dataset also included the 33 strikes from the lab experiment using western rattlesnakes (the 30 strikes used in the previous analysis, plus 3 strikes that were not paired), and 18 strikes from the field experiment using Mohave rattlesnakes. We found that whether snakes moved toward prey differed depending on the experimental context of the strike, both when the full dataset was used (χ^2^ = 46.27, df = 2, *P* < 0.0001, Fig. 3) and when we included only one strike per snake (χ^2^ = 27.02, df = 2, *P* < 0.0001). Post hoc comparisons showed that snakes striking under natural conditions were far less likely to move toward prey prior to striking compared to the lab experiment (both tests, *P* < 0.0001) and the field experiment (both tests, *P* < 0.0001), while the field experiment and lab experiment were not significantly different from each other (full dataset, *P* = 1; reduced dataset, *P* = 0.60).

## Discussion

Our results indicate that predatory strikes of rattlesnakes achieve similar maximum velocities across a range of temperatures. This result is most likely due to snakes not attempting to maximize attack velocity in predatory contexts: predatory strikes were not as rapid or explosive as defensive strikes, and defensive strikes reached higher maximum velocities and accelerations across all strike distances and temperatures. However, rattlesnakes do accelerate slightly faster at warmer temperatures, at least under some conditions. In addition, by comparing predatory strikes from three different contexts, we demonstrate that snakes striking free-ranging prey almost never exhibit the pre-strike head movements that are frequently seen toward artificial prey or in captivity—an apparent experimental artifact that has implications for the typical approaches used for measuring kinematic variables for striking snakes.

### Effects of temperature on strike performance

Previous studies indicate that rattlesnakes striking defensively achieve slightly faster maximum velocity and acceleration at warmer body temperatures (Rowe and Owings 1990; Stepp-Bolling 2012; Whitford et al. 2020). Our results suggest that predatory strikes are even less influenced by body temperature. In both the field and captive contexts, maximum strike acceleration was the only kinematic variable that was found to significantly increase with body temperature. Even though we did find a significant positive correlation between body temperature and maximum strike acceleration in the lab experiment, the effect was small—on average, maximum strike acceleration only increased by 19 m s^−2^ from 20°C to 30°C in the lab experiment, with temperature accounting for ∼34% of the variation in maximum strike acceleration. Similarly, body temperature only explained ∼23% of the variation in maximum strike acceleration in the field experiment. No kinematic measures were affected by temperature in our analysis of strikes from sidewinder rattlesnakes directed toward free-ranging kangaroo rats, a result potentially driven by snakes not prioritizing strike speed.

While it is unclear why the effects of temperature on predatory strikes are reduced compared to defensive strikes, there are several possible explanations. First, LaDuc (2002) documented significant kinematic and behavioral differences between predatory and defensive strikes, which may mediate the effects of temperature. Predatory strikes are typically initiated from a stereotyped coiled position, while the position of defensive snakes is highly variable, perhaps altering the ability of the snake to use elastic recoil to partially power strikes (Astley and Roberts 2014). However, this is speculative, as there have not been sufficiently detailed studies of the muscles used during strikes that would allow for more direct inferences about how body position and tendon recoil may interact with kinematics. Perhaps a more salient explanation for the difference between predatory and defensive strikes is in the motivations for the behaviors. Defensive movements are often more rapid than offensive ones. For example, prey capture events of northern pike (*Esox lucius*) exhibited significantly lower mean and maximum acceleration and velocity compared to escapes (Harper and Blake 1991). With defensive strikes, the snakes are trying to protect themselves and dissuade the predator from attacking. Like a boxer’s jab (Kimm and Thiel 2015), defensive strikes should pose a risk to the predator while limiting the snake’s own exposure to risk. Defensive strikes are likely also used as feints, or a means to keep a putative predator at a distance (Moon et al. 2019; Penning et al. 2019). In contrast, the purpose of the predatory strikes is to capture prey. This means snakes must target a relatively small moving target and approach it closely without causing it to startle prematurely—tasks that may be harder when moving quickly.

A recent analysis of the outcome of a series of predatory strikes from sidewinder rattlesnakes toward desert kangaroo rats found that the two biggest factors affecting the outcome were the accuracy of the strike (whether the snake’s head moved directly toward the body of the prey) and the reaction time of the kangaroo rat (Whitford et al. 2019b). Neither strike velocity nor acceleration was significantly associated with strike success. Because there is a general tradeoff in animal performance between speed and accuracy (Fitts 1954; Sinervo and Losos 1991; Jayne et al. 2014; Wheatley et al. 2015), we assume that this holds for snake strikes, but we do not know of any experimental studies that have addressed this.

There is also indirect evidence that the avoidance maneuvers of at least one common prey type, kangaroo rats, could be related to strike velocity and acceleration. Kangaroo rats appear to use their highly sensitive hearing to detect the low frequency sound made by a snake initiating a predatory strike (Webster 1962; Webster and Webster 1971). Although, again, experimental studies are lacking, it is possible that the volume of sound generated by a strike is related in part to the velocity and acceleration of the head and body. A similar dynamic has been studied extensively between field crickets and wolf spiders (Dangles et al. 2006b), with spiders running at high velocities creating a more detectable pattern of air disturbance and alerting crickets to their attack sooner than those moving at slower speeds. Perhaps rattlesnakes, like wolf spiders, moderate their speed, even when they could achieve higher velocities, in order to avoid triggering an early escape response. More research is needed to confirm or refute these hypotheses, but if rattlesnakes were not trying to move as fast as possible during predator strikes, then maximum velocity and acceleration would be less affected by temperature.

The temperature coefficients for predatory strikes also generally illustrate temperature independence. Q_10_ values for muscle-driven movements of ectotherms often show a doubling in performance (Q_10_ ≈ 2) for a 10°C increase in temperature (Bennett 1985; Herrel et al. 2007). However, rapid movements initiated from a standstill often illustrate a high robustness (Q_10_ ≈ 1) to changes in temperature (Anderson and Deban 2010; Deban and Scales 2016). While low temperature coefficients can indicate the presence of elastic recoil mechanisms, the reduced velocity of predatory strikes relative to defensive strikes suggests that the low Q_10_ values are the result of motivation and not elastic recoil. However, the Q_10_ values for defensive strikes are also lower than would be expected if the movement were driven purely by muscle contraction, indicating that the effects of temperature and motivation on the strike performance of both predatory and defensive strikes is complex and can only be resolved using more direct methods, such as *in situ* electromyography.

### Pre-strike head movements

With a growing body of literature illustrating that animals behave and perform differently in captive settings compared to naturalistic settings (Dangles et al. 2006a, Combes et al. 2012), we are sensitive to the fact that the predatory behaviors we are measuring in this study may not be entirely representative of the way in which snakes behave in nature. Due to the increasing use of field videography to study snake feeding ecology (Clark 2006; Barbour and Clark 2012b, Glaudas and Alexander 2016a, 2016b; Putman et al. 2016; Glaudas et al. 2017; Whitford et al. 2019a), we were able to notice qualitative differences in the predatory strike of rattlesnakes when in captivity or when striking an artificial target, such as a warmed mouse carcass (“non-natural” strikes). The clearest of these differences was the propensity of non-natural strikes to be preceded by a slow head movement toward the direction of the target, a behavior virtually absent for free-ranging snakes (Fig. 3) but reported frequently by other studies in captivity (Gillingham and Clark 1981; Young et al. 2001; Ryerson 2020). This movement has a substantial effect on the quantification of strike kinematics (Ryerson 2020). Almost all kinematic studies include the distance between the head of the snake and the target at the onset of the strike (typically referred to as strike distance) as an explanatory variable, and this distance often affects the maximum velocity and acceleration achieved by the strike (Herrel et al. 2011; Penning et al. 2019). As strike velocity is typically linearly related to strike distance, any change in distance will have a direct effect on the speed of a strike. The pre-strike head movement toward the prey frequently appears to reduce the strike distance by greater than 5 cm or more. While we do not have direct information as to why rattlesnakes exhibit this prestrike movement in our (and others’) experiments, it is more clear why they do not do so in nature: as ambush hunters, snakes would want to avoid any movement that might reveal their presence prior to striking. It is possible that moving toward novel items that are prey-like may be a behavior used to collect more information, allowing the snakes to assess whether they have correctly identified the novel item as prey. As it is unlikely that the snakes used in our field experiment had ever encountered a domestic mouse, they may have reacted this way because of the novel scent.

### Broader implications

Recent research has illustrated that asymmetries in responses to temperature in consumer–resource interactions may lead to changes in trophic interactions (Dell et al. 2011, 2014). The effects of asymmetric responses to temperature are predicted to be most pronounced in interactions between endothermic and ectothermic species due to the differences in body temperature and the correlation between temperature and locomotor performance. Rattlesnakes, and most vipers, interact with endothermic species both as predators and prey (Steenhof and Kochert 1985; Greene 1992; Hernández et al. 1994; Cartron et al. 2004). Although our research indicates that the outcome of predatory strikes toward small mammals may not be substantially influenced by changing environmental temperature. Additionally, defensive strikes of rattlesnakes are somewhat faster when snakes are warmer (Whitford et al. 2020), which indicates rattlesnakes could be more effective at defending themselves at higher temperatures, and thus experience some decreased predation risks in warming environments. It is likely that a much stronger effect of changing environmental temperature on predator–prey interactions would be driven by diel and seasonal activity patterns of rattlesnake foraging activity, as their willingness to hunt in exposed ambush coils exhibits clear thermal constraints (Clark et al. 2016; Putman and Clark 2017).

Our results are also suggestive that the predatory strategy of rattlesnakes may balance stealth and accuracy with speed, presumably in an attempt to subvert the evasive capabilities of prey. Future work on the sensory capabilities of small mammals, and the cues generated by the rattlesnake strike, would provide valuable insight into the potential for a stealth-speed tradeoff in the viperid sit-and-wait attack strategy.

## Supplementary Material

obaa025_Supplementary_Data
