# Supplementary material for: The Effects of Temperature on the Kinematics of Rattlesnake Predatory Strikes in Both Captive and Field Environments
Source: Integr Org Biol. 2020 Oct 4;2(1):obaa025. doi: 10.1093/iob/obaa025 (PMC7671150; doi:10.1093/iob/obaa025)
Supplement: obaa025_Supplementary_Data [file obaa025_supplementary_data.zip › 04212020_Supplementary Material.docx]

**Supplementary Material**

| 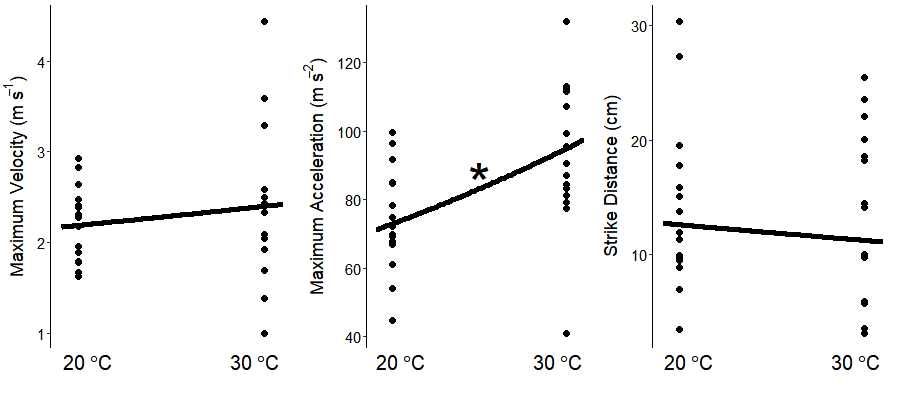 |
| --- |
| **Figure S1**: Scatterplots of maximum strike velocity, maximum strike acceleration, and strike distance for the lab predatory strikes of western rattlesnakes in the 20°C and 30°C treatments. Regression lines are back–transformed predictions from the mixed models. Asterisk (“*”) indicates a statistically significant relationship. |
